# Supplementary material for: Influenza virus uses mGluR2 as an endocytic receptor to enter cells
Source: Nat Microbiol. 2024 Jun 7;9(7):1764–77. doi: 10.1038/s41564-024-01713-x (PMC11222159; doi:10.1038/s41564-024-01713-x)
Supplement: Supplementary file 1 — Reporting Summary [file 41564_2024_1713_MOESM1_ESM.pdf]

nature portfolio

Corresponding author(s): Zhigao Bu and Hualan Chen

Last updated by author(s): Apr 12, 2024

Reporting Summary

Nature Portfolio wishes to improve the reproducibility of the work that we publish. This form provides structure for consistency and transparency in reporting. For further information on Nature Portfolio policies, see our Editorial Policies and the Editorial Policy Checklist.

Statistics

For all statistical analyses, confirm that the following items are present in the figure legend, table legend, main text, or Methods section.

n/a	Confirmed	
☐	☒	The exact sample size (*n*) for each experimental group/condition, given as a discrete number and unit of measurement
☐	☒	A statement on whether measurements were taken from distinct samples or whether the same sample was measured repeatedly
☐	☒	The statistical test(s) used AND whether they are one- or two-sided<br>*Only common tests should be described solely by name; describe more complex techniques in the Methods section.*
☒	☐	A description of all covariates tested
☒	☐	A description of any assumptions or corrections, such as tests of normality and adjustment for multiple comparisons
☐	☒	A full description of the statistical parameters including central tendency (e.g. means) or other basic estimates (e.g. regression coefficient) AND variation (e.g. standard deviation) or associated estimates of uncertainty (e.g. confidence intervals)
☐	☒	For null hypothesis testing, the test statistic (e.g. *F*, *t*, *r*) with confidence intervals, effect sizes, degrees of freedom and *P* value noted<br>*Give P values as exact values whenever suitable.*
☒	☐	For Bayesian analysis, information on the choice of priors and Markov chain Monte Carlo settings
☒	☐	For hierarchical and complex designs, identification of the appropriate level for tests and full reporting of outcomes
☒	☐	Estimates of effect sizes (e.g. Cohen's *d*, Pearson's *r*), indicating how they were calculated

Our web collection on statistics for biologists contains articles on many of the points above.

Software and code

Policy information about availability of computer code

Data collection	Images and histochemistry images were acquired by using a confocal laser scanning microscope 980 and 700, STED images were taken by using a Abberior instruments stedycon equipped with an IX83 microscope. Electron microscope images of the process of influenza virus internalization were taken by using Hitachi-7650 transmission electron microscope.
Data analysis	GraphPad Prism (version 8.0) was used for data statistical analysis; ZEN software (version 2.3) was used for calculation of fluorescence intensity of images; FlowJo (version 10.0) was used for flow cytometry assay; Image J (version 1.53t) was used to analyze the intensity of protein bands. Stedycon Smart Control software (firmware version 9.0.696-g982382b; FPGA version 13-g2becb89; headboard firmware version 2.11) was used to convert the STED images.

For manuscripts utilizing custom algorithms or software that are central to the research but not yet described in published literature, software must be made available to editors and reviewers. We strongly encourage code deposition in a community repository (e.g. GitHub). See the Nature Portfolio guidelines for submitting code & software for further information.

Data

Policy information about availability of data

All manuscripts must include a data availability statement. This statement should provide the following information, where applicable:

- Accession codes, unique identifiers, or web links for publicly available datasets
- A description of any restrictions on data availability
- For clinical datasets or third party data, please ensure that the statement adheres to our policy

The authors declare that the data supporting the findings of this study are available within the paper and its source data files. The mRNA sequencing data of mGluR2 (Extended Data Fig. 3) in human tissues was from BioGPS database (http://biogps.org/#goto=genereport&id=2912).

Research involving human participants, their data, or biological material

Policy information about studies with human participants or human data. See also policy information about sex, gender (identity/presentation), and sexual orientation and race, ethnicity and racism.

Reporting on sex and gender	not applicable
Reporting on race, ethnicity, or other socially relevant groupings	not applicable
Population characteristics	not applicable
Recruitment	not applicable
Ethics oversight	not applicable

Note that full information on the approval of the study protocol must also be provided in the manuscript.

Field-specific reporting

Please select the one below that is the best fit for your research. If you are not sure, read the appropriate sections before making your selection.

☒ Life sciences ☐ Behavioural & social sciences ☐ Ecological, evolutionary & environmental sciences

For a reference copy of the document with all sections, see nature.com/documents/nr-reporting-summary-flat.pdf

Life sciences study design

All studies must disclose on these points even when the disclosure is negative.

Sample size	For all experiments, 3 biological independent experiments for statistical relevance (the unpaired, two-tailed Student's t-test).<br>For mice experiments, 18 mice per group, more than the number of mice usually used for this type of experiment (8 mice).
Data exclusions	No data were excluded from the analyses.
Replication	3 independent experiments, consistent data between the experiment, all replication attempts were successful.
Randomization	For in vitro study, randomization is not possible because the same cells were treated differently, therefore a random distribution of the samples was not possible. For in vivo study, randomization was not possible because the mice in two groups were genetically different.
Blinding	For all experiments, the samples were not blinded but the analysis were blinded (the experimenters that run the experiments and the one that analyzed the data were different, any information of the samples was not showed during the process of analysis).

Reporting for specific materials, systems and methods

We require information from authors about some types of materials, experimental systems and methods used in many studies. Here, indicate whether each material, system or method listed is relevant to your study. If you are not sure if a list item applies to your research, read the appropriate section before selecting a response.

Materials & experimental systems

n/a	Involved in the study
☐	☒ Antibodies
☐	☒ Eukaryotic cell lines
☒	☐ Palaeontology and archaeology
☐	☒ Animals and other organisms
☒	☐ Clinical data
☒	☐ Dual use research of concern
☒	☐ Plants

Methods

n/a	Involved in the study
☒	☐ ChIP-seq
☐	☒ Flow cytometry
☒	☐ MRI-based neuroimaging

Antibodies

Antibodies used

Rabbit anti-H1-hemagglutinin polyclonal antibody (pAb), Sino Biological, Cat# 11692-T62, microscopy-based assay, 1:300
Rabbit anti-Flag-tag pAb, Genscript, Cat# A00170, western blotting assay, 1:1000
Rabbit anti-Myc-tag pAb, Genscript, Cat# A00172, western blotting assay, 1:1000; immunoelectron microscopy assay, 1:50
Rabbit anti-AQP5 pAb antibody, Boster, Cat# A03085, immunofluorescence histochemistry, 1:100
Rabbit anti-6-His pAb, Sigma-Aldrich, Cat# SAB4301134, western blotting assay, 1:1000
Goat anti-chicken IgY coupled with HRP, Sigma-Aldrich, Cat# A9046, western blotting assay, 1:2000
Horse anti-mouse IgG coupled with HRP, Vectorlabs, Cat# MP-7802-15, microscopy-based assay, immunofluorescence histochemistry, 1:5
Goat anti-rabbit IgG coupled with gold (10 nm), Sigma-Aldrich, Cat# G7402, immunoelectron microscopy assay, 1:1000
Goat anti-mouse IgG coupled with Alexa Fluor 568, Abcam, Cat# ab175473, microscopy-based assay, 1:300
Goat anti-mouse IgG coupled with Alexa Fluor 488, Abcam, Cat# ab150113, microscopy-based assay, 1:300
Goat anti-rabbit IgG coupled with Alexa Fluor 488, Invitrogen, Cat# A11034, microscopy-based assay, 1:300
Goat anti-rabbit IgG coupled with HRP, Invitrogen, Cat# 31460, western blotting assay, 1:2000
Goat anti-mouse Abberior STAR RED, Abberior, Cat# 20831PK-3, STED Microscopy assay, 1:200,
Goat anti-mouse Abberior STAR ORANGE, Abberior, Cat# 20831PK-6, STED Microscopy assay, 1:200
Goat anti-mouse IgG coupled with Cy3, Beyotime, Cat# A0521, immunofluorescence histochemistry, 1:100
Goat anti-rabbit IgG coupled with fluorescein, Vectorlabs, Cat# FI-1000, immunofluorescence histochemistry, 1:200
Chicken anti-H5 or H7-hemagglutinin pAb, produced in our lab, western blotting assay, 1:500
Rabbit anti-KCa1.1 pAb antibody, Alomone Labs, APC-151, microscopy-based assay, 1:100
Rabbit anti-MUC1 pAb antibody, Bioss, Cat# bs-1497R, immunofluorescence histochemistry, 1:100
Mouse anti-mGluR2 monoclonal antibody (mAb) [A-1], Santa Cruz Biotechnology, Cat# sc271654, microscopy-based assay,1:200
Mouse anti-nucleoprotein mAb antibody [10E9], produced in our lab, immunofluorescence histochemistry, 1:200
Mouse IgG2a mAb [HOPC-1], Southern Biotech, Cat# 0103-01, antibody blocking assay, 1:25
Rabbit anti-Na+/K+ ATPase mAb [EP1845Y], Abcam, Cat# ab76020, western blotting assay, 1:500
Rabbit anti-S100A9 mAb antibody [EPR22332-75], Abcam, Cat# ab242945, immunofluorescence histochemistry, 1:800
Rabbit anti-V5-tag mAb [D3H8Q], Cell Signaling, Cat# 13202S, western blotting assay, 1:1000

Validation

All the commercial antibodies have been verified by the manufactures from their websites. Chicken anti-H5 or H7-hemagglutinin pAbs were validated by Yuancheng Zhang (PMID: 38005926) and Xin Yin (PMID:33905456). Mouse anti-nucleoprotein mAb antibody [10E9] was validated by Yuhui Zhao (DOI:10.1016/S2095-3119(21)63840-6).

Eukaryotic cell lines

Policy information about cell lines and Sex and Gender in Research

Cell line source(s)

HEK293 cells (ATCC, CRL-1573), A549 cells (ATCC, CCL-185), MDCK cells (ATCC, PTA-6500), 293E cells (ATCC, CRL-1573) were purchased from ATCC

Authentication

All cells used in this study were verified by ATCC. Cell morphology was monitored at each passage by microscope. We will discard the cells after 15 passages, and recover new cells from frozen stocks.

Mycoplasma contamination

All cell lines tested negative for mycoplasma contamination.

Commonly misidentified lines
(See ICLAC register)

No commonly misidentified lines were used in this study.

Animals and other research organisms

Policy information about studies involving animals; ARRIVE guidelines recommended for reporting animal research, and Sex and Gender in Research

Laboratory animals

Six-week-old wild-type and mGluR2 gene knockout C57BL/6J mice were used in this study. Mice were housed in ventilated cages (up to 8 mice/cage) with food and water ad libitum. Mice were housed under 12 h light/dark cycles in a controlled environment maintained at 24 degreeCelsius and 40-60% humidity.

Wild animals	This study did not involve wild animals.
Reporting on sex	Male and female mice were both used in this study.
Field-collected samples	This study did not involve samples collected from field.
Ethics oversight	Animal studies were carried out in strict accordance with the recommendations in the Guide for the Care and Use of Laboratory Animals of the Ministry of Science and Technology of the People's Republic of China. The protocols were approved by the Committee on the Ethics of Animal Experiments of Harbin Veterinary Research Institute (HVRI) of Chinese Academy of Agricultural Sciences (CAAS) (approval number IACUC-2022-220719-01-GJ).

Note that full information on the approval of the study protocol must also be provided in the manuscript.

Plants

Seed stocks	not applicable
Novel plant genotypes	not applicable
Authentication	not applicable

Flow Cytometry

Plots

Confirm that:

☒ The axis labels state the marker and fluorochrome used (e.g. CD4-FITC).

☒ The axis scales are clearly visible. Include numbers along axes only for bottom left plot of group (a 'group' is an analysis of identical markers).

☒ All plots are contour plots with outliers or pseudocolor plots.

☒ A numerical value for number of cells or percentage (with statistics) is provided.

Methodology

Sample preparation	A549 cells were transfected with pmGluR2 or pmGluR2-mutant for 24 h and collected in a 1.5-mL tube. Then the cells were washed three times with FACS wash buffer (PBS containing 2% FCS) and fixed with 4% PFA at room temperature for 15 min. The cells were then permeabilized with 0.5% Triton X-100 for 30 min (skip this step in the unpermeabilized condition). The permeabilized or unpermeabilized cells were incubated with mouse anti-mGluR2 mAb (1:100) as the primary antibody and goat anti-mouse IgG coupled with Alexa Fluor 488 (1:1000) as the secondary antibody. The cells were analyzed by using a FC-500 flow cytometer (Beckman Coulter). The fluorescence intensity of mGluR2 was analyzed by using Flow Jo software.
Instrument	FC500 flow cytometer (Beckman Coulter).
Software	FlowJo (version 10.0)
Cell population abundance	N/A. Sorting was not performed so not applicable here.
Gating strategy	Flow cytometric analyses were gated by FSC, SSC scatters. See diagram of strategy in Source Data.

☒ Tick this box to confirm that a figure exemplifying the gating strategy is provided in the Supplementary Information.
