# Supplementary material for: Influenza virus uses mGluR2 as an endocytic receptor to enter cells
Source: Nat Microbiol. 2024 Jun 7;9(7):1764–77. doi: 10.1038/s41564-024-01713-x (PMC11222159; doi:10.1038/s41564-024-01713-x)

Fig. 2e, Interaction of mGluR2 and KCa1.1 confirmed by co-immunoprecipitation with the anti-Flag antibody coupled agarose beads

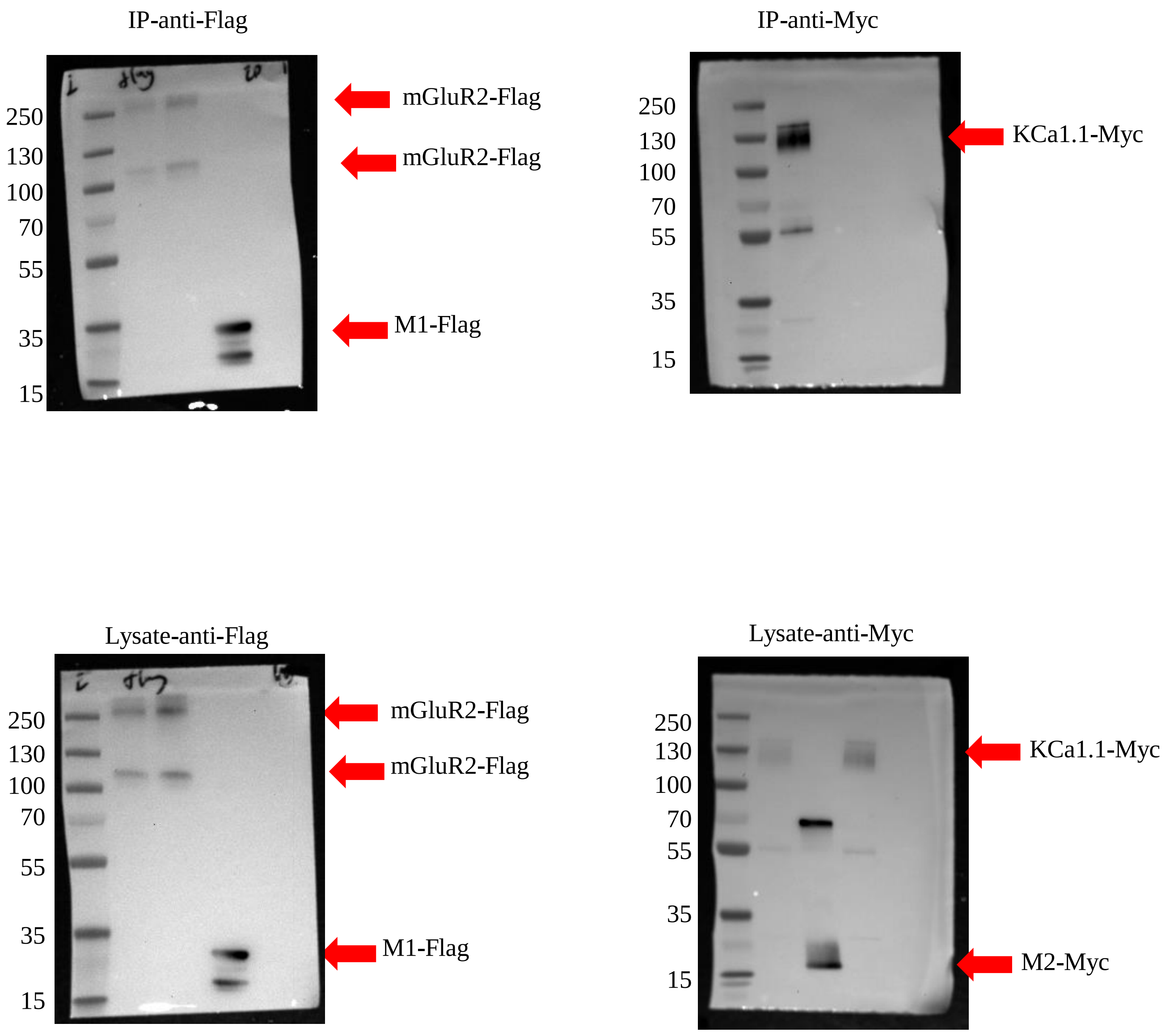

Supplement: Supplementary file 6 — Unprocessed western blots. [file 41564_2024_1713_MOESM6_ESM.pdf]
