# Supplementary material for: Influenza virus uses mGluR2 as an endocytic receptor to enter cells
Source: Nat Microbiol. 2024 Jun 7;9(7):1764–77. doi: 10.1038/s41564-024-01713-x (PMC11222159; doi:10.1038/s41564-024-01713-x)

Fig. 3b, Direct interaction of mGluR2 and HA demonstrated by use of a pull-down assay with the anti-Flag antibody coupled agarose beads.

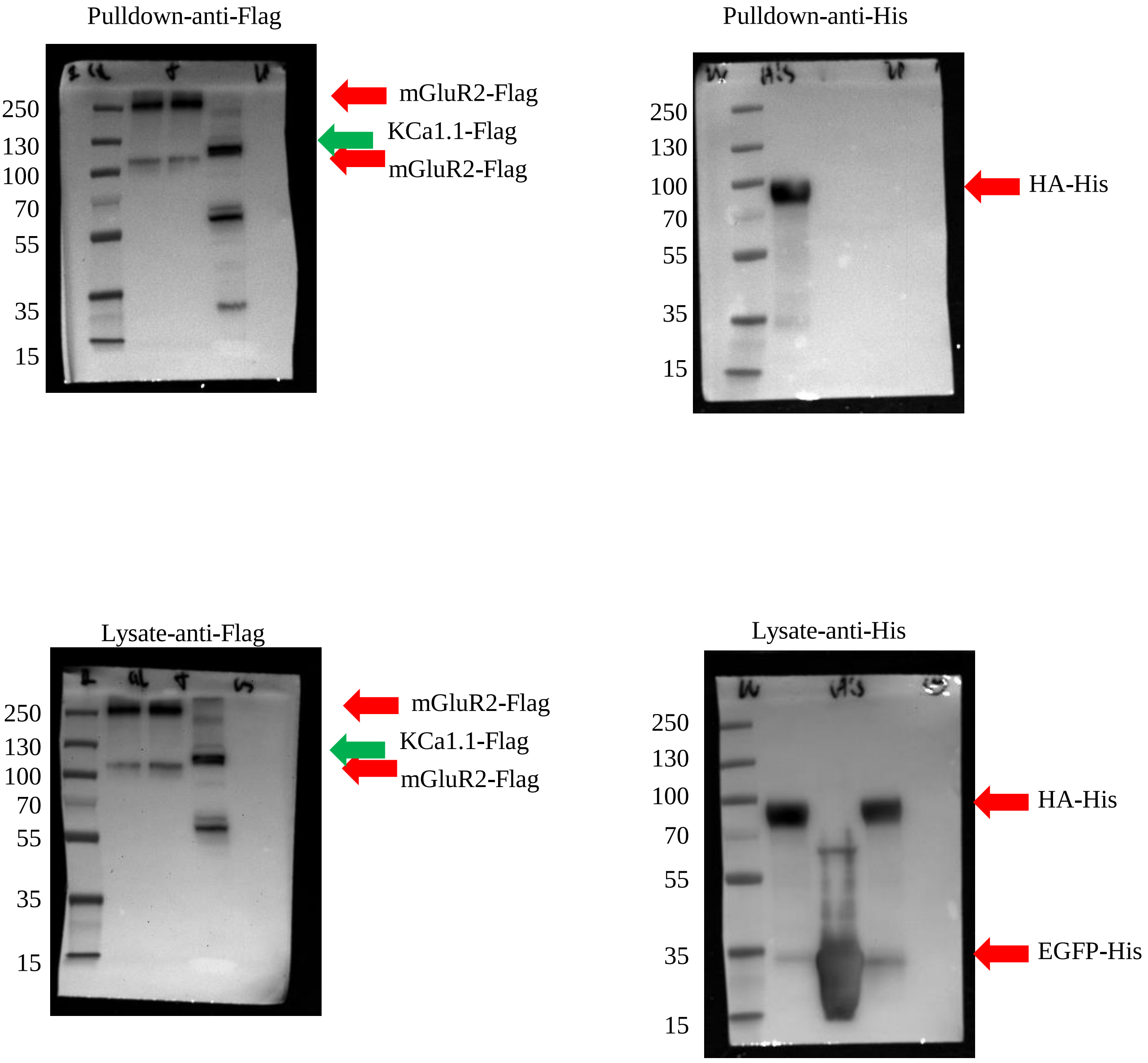

Supplement: Supplementary file 8 — Unprocessed western blots. [file 41564_2024_1713_MOESM8_ESM.pdf]
