# Supplementary material for: Influenza virus uses mGluR2 as an endocytic receptor to enter cells
Source: Nat Microbiol. 2024 Jun 7;9(7):1764–77. doi: 10.1038/s41564-024-01713-x (PMC11222159; doi:10.1038/s41564-024-01713-x)

Extended Data Fig. 2c, KCa1.1 does not interact with HA. Direct interaction of KCa1.1 and HA was tested by use of a pull-down assay with the anti-Myc antibody coupled agarose beads.

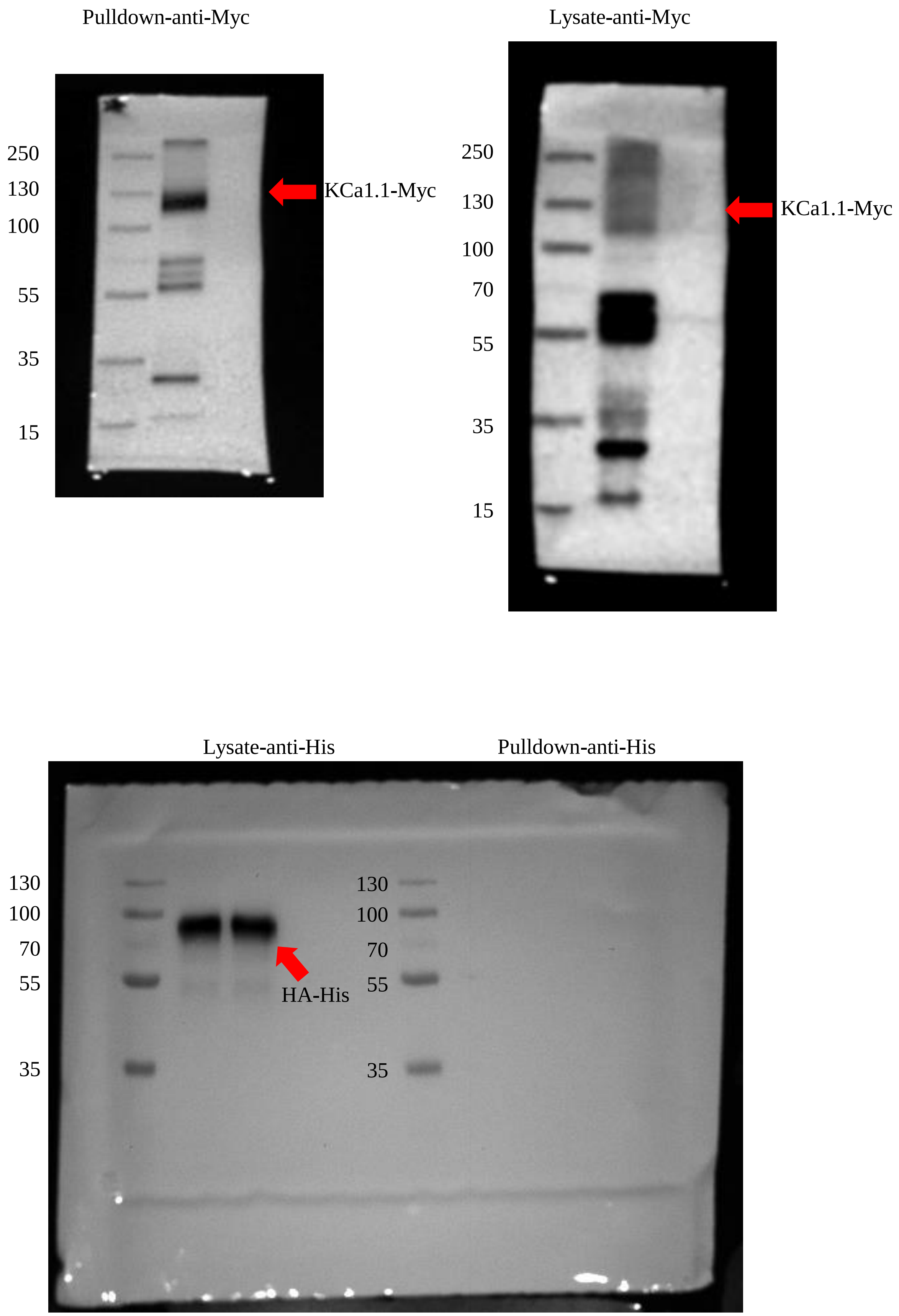

Supplement: Supplementary file 12 — Unprocessed western blots. [file 41564_2024_1713_MOESM12_ESM.pdf]
