# Supplementary material for: Influenza virus uses mGluR2 as an endocytic receptor to enter cells
Source: Nat Microbiol. 2024 Jun 7;9(7):1764–77. doi: 10.1038/s41564-024-01713-x (PMC11222159; doi:10.1038/s41564-024-01713-x)

Extended Data Fig. 8b, Interaction between viral HA and mGluR2 or mGluR2-mutant was analyzed by using co-immunoprecipitation with the anti-Flag antibody coupled agarose beads.

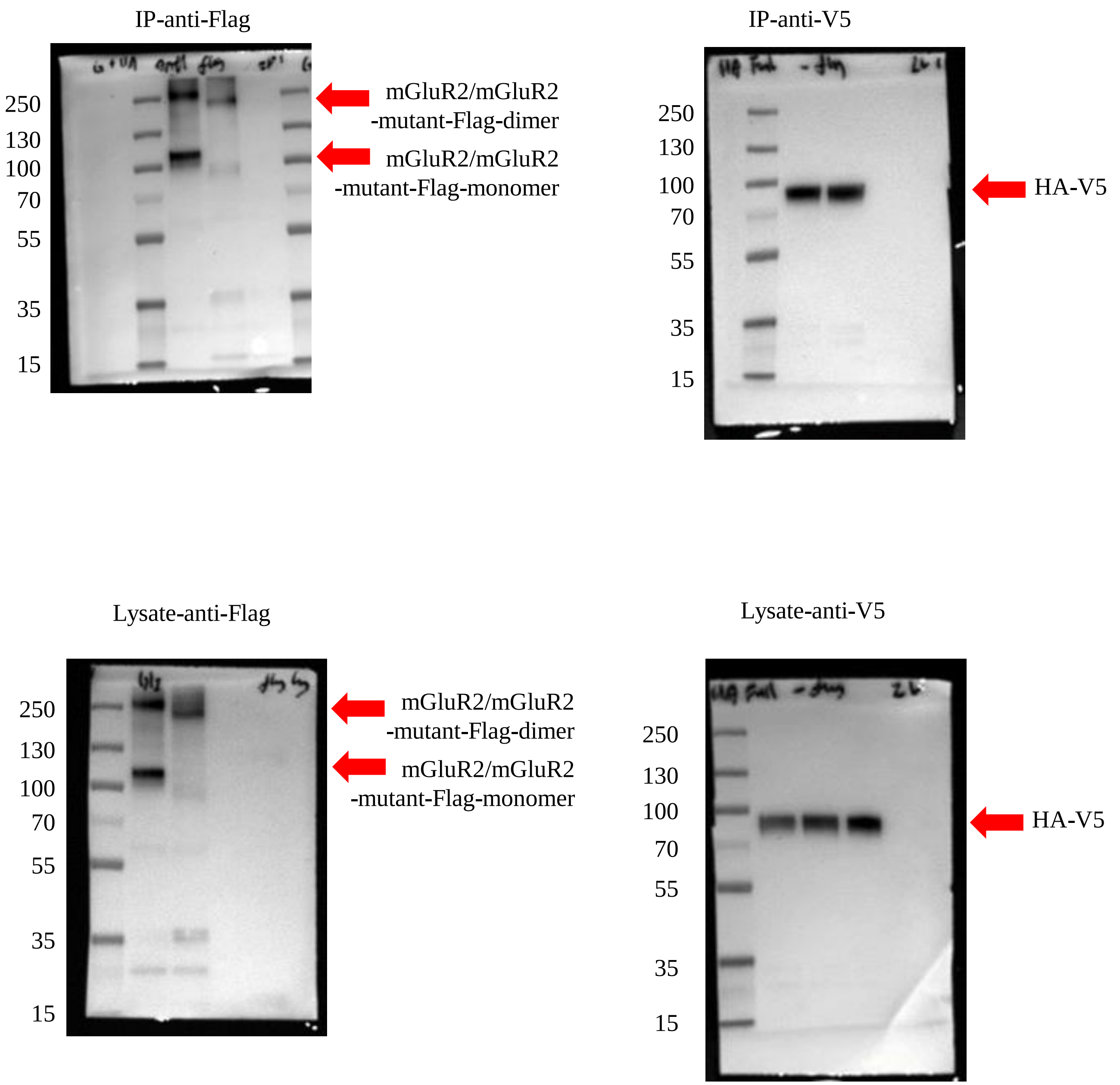

Extended Data Fig. 8c, Interaction between viral HA and mGluR2 or mGluR2-mutant was analyzed by using pulldown assay with the anti-Flag antibody coupled agarose beads.

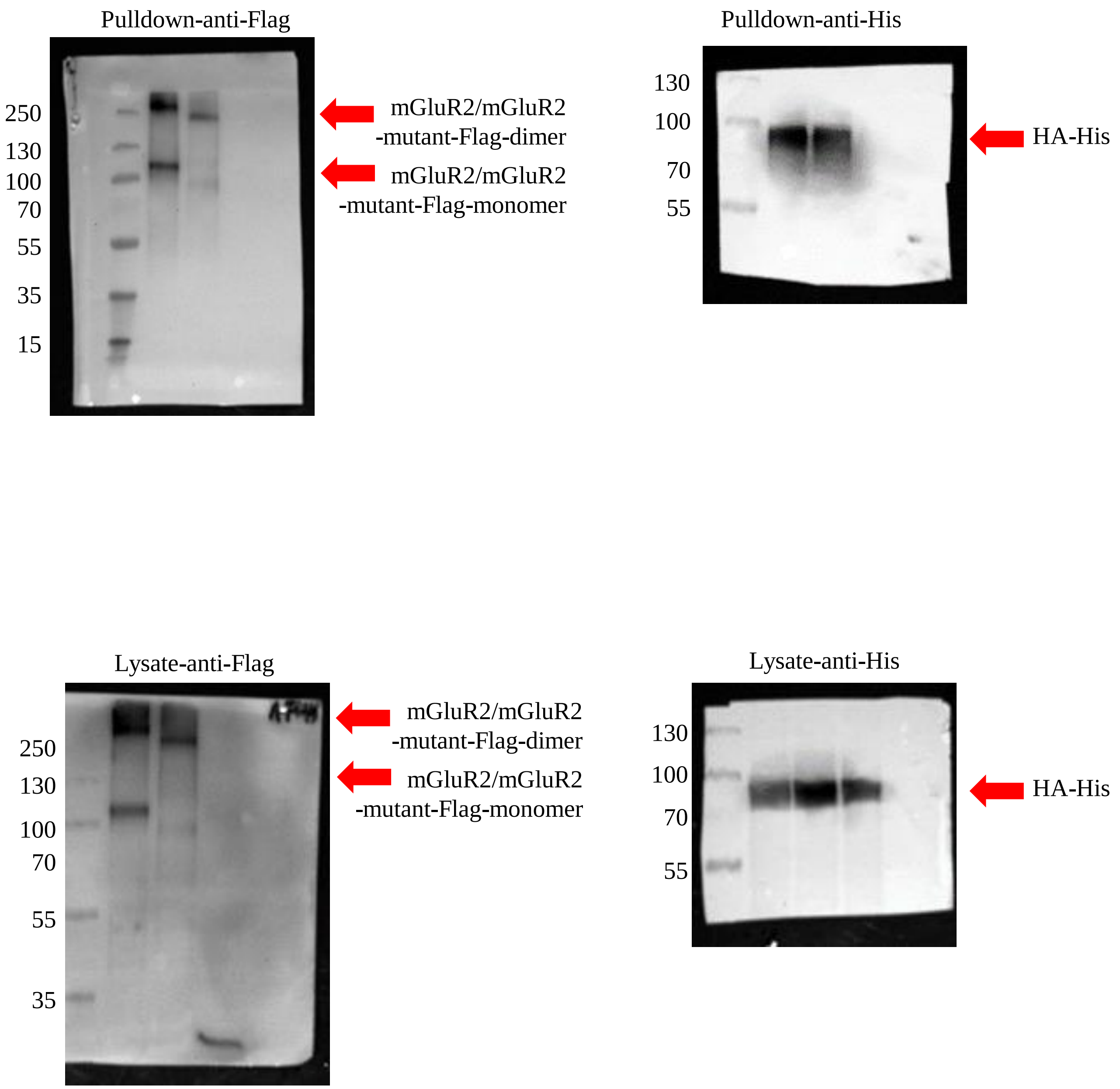

Extended Data Fig. 8e, The abundance of mGluR2 and mGluR2-mutant in whole cells and on the plasma membrane confirmed by western blotting

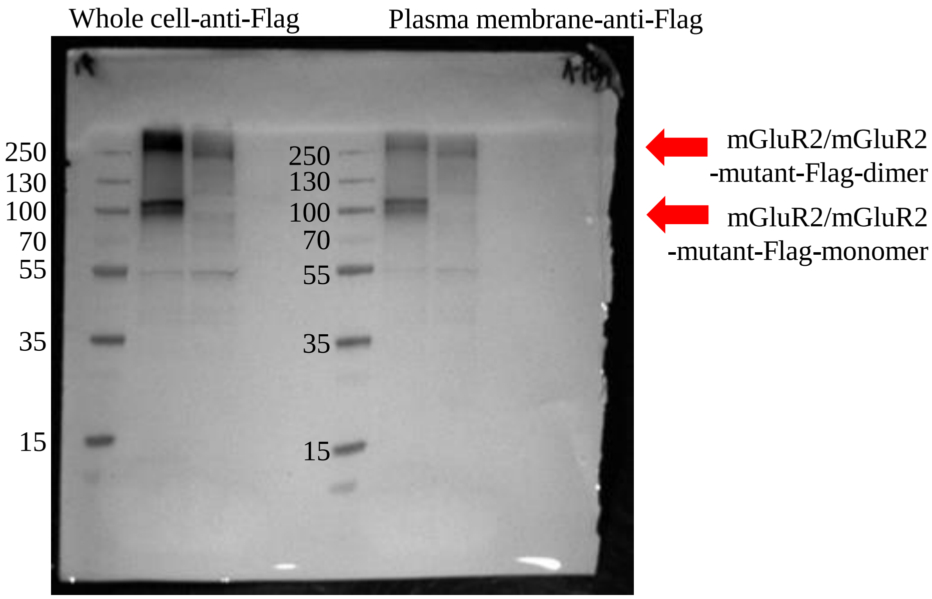

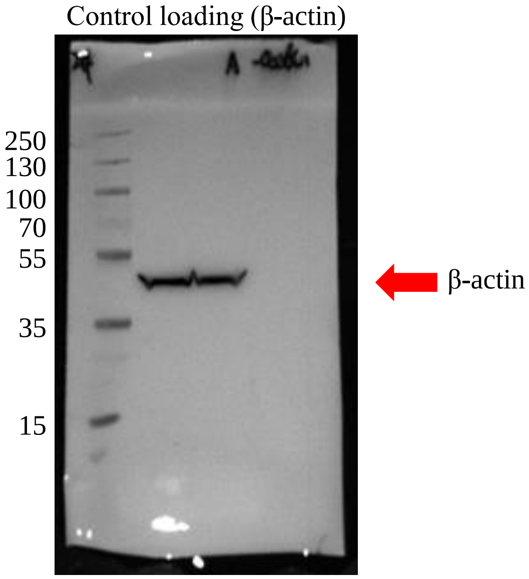

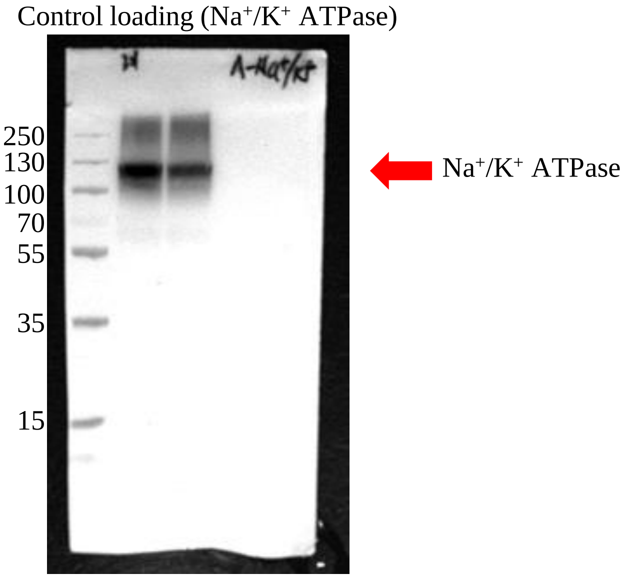

Supplement: Supplementary file 18 — Unprocessed western blots. [file 41564_2024_1713_MOESM18_ESM.pdf]
